# Reduced Sensitivity of Influenza A (H5N1) to Oseltamivir

**DOI:** 10.3201/eid1309.07-0164

**Published:** 2007-09

**Authors:** Jennifer L. McKimm-Breschkin, Paul W. Selleck, Tri Bhakti Usman, Michael A. Johnson

**Affiliations:** * CSIRO Molecular and Health Technologies, Parkville, Victoria, Australia; † CSIRO Livestock Industries, Geelong, Victoria, Australia; ‡ Disease Investigation Centre Region IV, Yogyakarta, Indonesia

**Keywords:** Avian influenza, H5N1, oseltamivir, zanamivir, antiviral drug resistance, neuraminidase, dispatch

## Abstract

We tested the neuraminidase drug sensitivity of clade 1 and clade 2 influenza A virus (H5N1). All viruses demonstrated similar sensitivity to zanamivir, but compared to the 2004 clade 1 viruses, the Cambodian 2005 viruses were 6-fold less sensitive and the Indonesian clade 2 viruses were up to 30-fold less sensitive to oseltamivir.

Two different strains of highly pathogenic avian influenza A (H5N1) have been circulating since 2003. Clade 1 has been found in Vietnam, Thailand, Cambodia, Lao People’s Democratic Republic, and Malaysia. Clade 2 subsequently emerged and spread from People’s Republic of China to Indonesia, Europe, and Africa in 2004–2005. Because of its systemic availability, oseltamivir is the drug of choice for treating infected persons (*1*).

## The Study

We tested the drug sensitivity of neuraminidases (NAs) (*2*) from influenza (H5N1) from chickens, ducks, geese, and quail^1^ from 2004 from Vietnam and Malaysia (provided by N. Long, Regional Animal Health Centre, Ho Chi Minh City, Vietnam; and S. Hassan, Veterinary Research Institute, Ipoh, Malaysia), from 2004–05 from Cambodia (provided by S. San, National Animal Health Production and Investigation Center, Phnom Penh, Cambodia), and from 2005 from Indonesia (provided by T. Usman). In the absence of a validated cell culture assay, the 50% inhibitory concentration measured in the NA enzyme inhibition assay is used as the benchmark for measuring drug sensitivity (*3*).

Despite their origins in different countries and different avian species, all clade 1 and clade 2 viruses had a similar sensitivity to zanamivir as the reference influenza (H1N1) NA, (Table 1, Figure, panel A). However, sensitivities of the NAs to oseltamivir (oseltamivir carboxylate) fell into 3 groups when compared to the NA of a reference human influenza (H1N1) strain (Table 1, Figure, panel B). The clade 1 isolates from 2004 were all more sensitive to oseltamivir than was the human influenza (H1N1) control, consistent with recent findings of Rameix-Welti et al. (*4*). However, the NAs of our 2005 Cambodian viruses showed a 6- to 7-fold decrease specifically in oseltamivir sensitivity in comparison to our 2004 Cambodian isolates. These 2005 isolates came from the same area as 1 of the more sensitive 2004 isolates (Kandal), which suggested that at least regional evolution had occurred.

**Table 1:** Mean IC_50_s of NA sensitivities in MUNANA*-based enzyme inhibition assay for influenza (H5N1) isolates from each region compared with a human influenza (H1N1) control and known resistant H274Y isolate†

Isolate	Zanamivir,‡§ mean IC_50_, nmol/L	Oseltamivir,‡§ mean IC_50_, nmol/L	4-Amino-Neu5Ac2en,‡§ mean IC_50_, μmol/L
Subtype H1N1			
A/Mississippi/3/2001 [2]¶ wt	1.18 (0.24)	2.16 (0.31)	1.12†
A/Mississippi/3/2001 H274Y [4]¶	1.41(0.26)	475.1 (344)	1.31†
Clade 1 subtype H5N1 2004			
Malaysia 2004 [2]	1.21 (0.13)	0.47 (0.07)	2.82 (0.77)
Vietnam 2004 [8]	1.40 (0.44)	0.55 (0.26)	2.47 (0.42)
Cambodia 2004 [6]	1.96 (0.56)	0.41 (0.24)	2.59 (0.15)
Clade 1 subtype H5N1 2005			
Cambodia 2005 [4]	1.53 (0.4)	2.88 (0.58)	2.56 (0.53)
Clade 2 subtype H5N1			
Indonesia 2005 [6]	1.42 (0.63)	11.45 (4.32)	2.00 (0.77)

*Sigma, Saint Louis, MO, USA. †IC_50_, drug concentration for 50% inhibition of enzyme activity; NA, neuraminidase; wt, wild type. ‡Drugs provided by GlaxoSmithKline (Stevenage, Hertfordshire, UK). §Nos. in brackets indicate the nos. of isolates tested against zanamivir and oseltamivir in the same assay. Nineteen influenza (H5N1) isolates were retested in an independent assay against oseltamivir and 4-amino-Neu5Ac2en. Means for oseltamivir are the results of the 2 independent experiments. IC_50_s for each isolate were calculated by using the Sigmaplot nonlinear curve-fitting function (Systat Software Inc., London, UK). Values in parentheses are standard deviations for the means of the individual IC_50_s for the isolates in each group. ¶Several plaques were tested from the A/Mississippi/3/2001 against zanamivir and oseltamivir, but only 1 plaque of each was tested against 4-amino-Neu5Ac2en.

**Figure:**
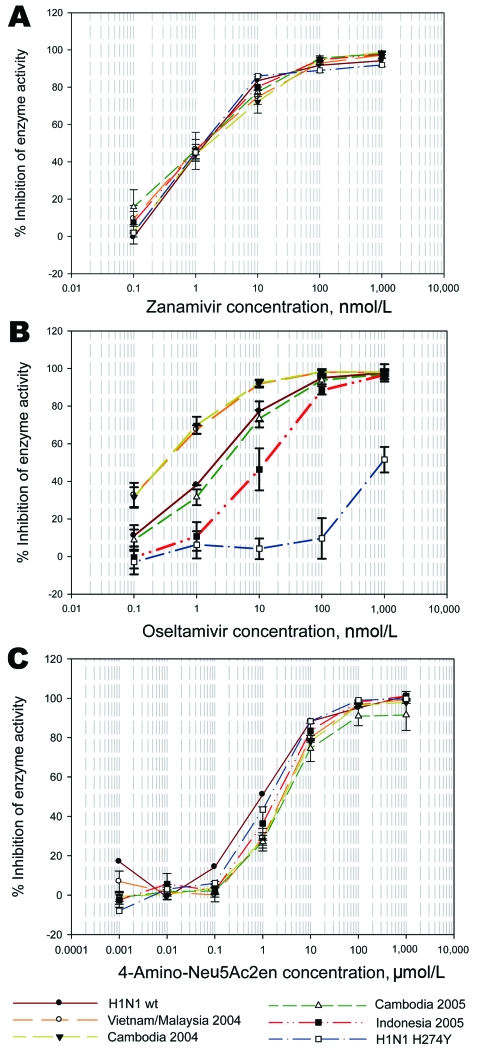
Sensitivity of clade 1 and clade 2 influenza A (H5N1) viruses to zanamivir, oseltamivir, and 4-amino-Neu5Ac2en in a MUNANA-based enzyme inhibition assay (Sigma, Saint Louis, MO, USA). Viruses were grown in allantoic fluid and irradiated for testing sensitivities of their neuraminidases. Plots are the mean values for inhibition of enzyme activity for each drug concentration of all isolates from that country and year; bars represent standard deviations of values for all isolates from that group. A) Sensitivity to zanamivir; B) sensitivity to oseltamivir; C) sensitivity to 4-amino-Neu5Ac2en.

Of more concern was the third group. The NAs from all the clade 2 2005 Indonesian viruses demonstrated a 15- to 30-fold decrease in sensitivity specifically to oseltamivir compared with clade 1 viruses (Table 1, Figure, panel B). Govorkova et al. (*5*) also recently showed that the A/Turkey/15/2006 clade 2 virus was almost 60-fold less sensitive to oseltamivir in a plaque reduction assay than was the clade 1 A/Vietnam/1203/2004 virus. Both these results and those of Rameix-Welti et al. (*4*) contrast to those recently published by Hurt et al., who found no difference between the sensitivities of clade 1 and clade 2 isolates to oseltamivir in the enzyme assay (*6*), although many of the isolates were the same as those tested here. The reason for the discrepancy is not known. The decrease in sensitivity is comparable to that conferred by the N294S recently detected in Egypt (*7*), known to be selected for by oseltamivir treatment (*8*).

Although both drugs are based on the transition state analog of sialic acid, zanamivir has a single substitution of a guanidinium group at the 4′ position on the sugar ring, whereas oseltamivir has an amino group at the 4′ position and, more importantly, a bulky hydrophobic pentyl ether group replacing the glycerol side chain at the 6′ position. Reorientation of E276 in the active site is required to create a hydrophobic pocket necessary to accommodate this pentyl ether group. Mutations that prevent this reorientation from occurring lead to high levels of specific oseltamivir resistance (H274Y, R292K). Decreased binding to oseltamivir can also be due to altered interactions with its 4-amino group (E119V) (*9*). We therefore tested several viruses from each group for inhibition by a drug that shares the 4-amino substitution. The clade 1 and 2 viruses had similar sensitivity to 4-amino-Neu5Ac2en (Table 1, Figure, panel C), indicating the decreased binding to oseltamivir was specifically due to altered interactions around the 6-pentyl ether group, thus explaining why no altered binding to zanamivir was seen.

Because of the potentially important implications of our findings for public health and stockpiling strategies, and because of the apparent discrepancy with the results of Hurt et al. (*6*), we retested 2 viruses from each group against an independent source of oseltamivir carboxylate (provided by Biota, Notting Hill, Victoria, Australia). We chose A/Indonesia/Wates/77/2005, which had a further slight decrease in sensitivity compared to other Indonesian isolates, as well as one that was in the same range as the remaining Indonesian isolates. Results shown in Table 2 confirm this decreased sensitivity of the clade 2 Indonesian isolates and a small further decrease in sensitivity of A/Indonesia/Wates/77/2005.

**Table 2:** Mean IC_50_s of isolates from each group against alternative source of oseltamivir*†

Virus	Oseltamivir, IC_5_**_0_**, nmol/L
A/Mississippi/3/2001 wt	3.2 (2.1)
A/Chicken/Vietnam/008/2004	0.7 (0.4)
A/Chicken/Cambodia/Kandal/23/2004	0.4 (0.2)
A/Goose/Cambodia/Kandal/2005	4.2 (0.5)
A/Chicken/Cambodia/Kandal/3/2005	4.8 (0.5)
A/Chicken/Indonesia/Wates/77/2005	25.6 (2.5)
A/Chicken/Indonesia/Wates/126/2005	14.7 (2.3)

*IC_50_, drug concentration for 50% inhibition of enzyme activity; wt, wild type. †Isolates were tested in duplicate in 2 separate assays. IC_50_s and standard errors (in parentheses) were calculated by using the nonlinear curve-fitting function in Sigmaplot (Systat Software Inc., London, UK).

Since we only had access to clade 2 isolates from Indonesia, we do not know whether this decreased sensitivity occurs only in the Indonesian clade 2 isolates or globally with all clade 2 isolates. Comparisons of the sequences in the public databases show several mutations in the stalk region, which vary between clade 1 and clade 2 NAs, but 1 mutation in the globular head, H252Y, varies between all clade 1 and clade 2 isolates. A further 3-aa variation occurs in most clade 1 and clade 2 NAs, S343P, E387G, and G459S (N2 numbering), including the Indonesian isolates studied here. An additional V338M variation is found in most Indonesian isolates, including the ones studied here (sequences submitted by N. Komadina). Whether this additional variation confers this decreased oseltamivir sensitivity remains to be elucidated. The A/Indonesia/Wates/77/2005 NA has an additional unique I117V variation, which is adjacent to one of the catalytic arginines, R118 (which would be consistent with a further effect on drug sensitivity to oseltamivir), and a slight decrease in sensitivity to zanamivir (2.3 nmol/L vs mean of 1.4 nmol/L for other Indonesian isolates).

## Conclusions

We have shown here that, compared with clade 1 isolates from 2004, some clade 1 Cambodian isolates and clade 2 Indonesian isolates from 2005 demonstrate reduced sensitivity to oseltamivir. Because none of the sequence variations in the public databases correlates with any mutation known to confer oseltamivir resistance, and none of the variations are in the active site (*10*), this suggests that the decrease in sensitivities may be due to drift mutations rather than from exposure to oseltamivir. Recent results show that human isolates can also demonstrate decreased sensitivity to oseltamivir and zanamivir with drift mutations in the NA remote from the active site (*11*). The difference in the amino acid at position 252 may partially account for the altered binding between the clade 1 and clade 2 NAs as other researchers have suggested that the amino acid at position 252 can affect reorientation of the E276 (*4*,*10*). In clade 1 NAs, this is normally subtype H252, but all clade 2 NAs have subtype Y252. In the recently published N1 structure, although it is from a clade1 subtype H5N1 NAs, the authors had mutated the NA to subtype Y252 (*10*), which is also found in human NAs. The E276 in this structure does not appear to have undergone full reorientation, which could contribute to reduced oseltamivir binding found in the Indonesian clade 2 isolates here. Testing the sensitivities of both NAs would provide valuable information on the role of subtype H252. However, mutational analysis of the Indonesian and Cambodian isolates and structure determinations of the different NAs are necessary to fully understand the mechanisms of altered binding.

The specific decrease in sensitivity to oseltamivir of both 2005 Cambodian clade 1 and especially the Indonesian clade 2 influenza (H5N1) isolates is disturbing, especially since they maintain their pathogenicity and transmissibility in birds and are clearly pathogenic in humans, since Indonesia has the highest death rate from influenza (H5N1) infections of any country. This finding is in contrast to recent observations that mutations conferring zanamivir resistance in human strains have poor viability and are not genetically stable (*12*). Such a decrease in oseltamivir sensitivity could lead to suboptimal drug dosing in treating persons infected with these isolates, which is thought to facilitate selection of viruses with a high level of resistance (*13*). Several groups have reported the emergence of resistant viruses in clade 1–infected influenza (H5N1) patients treated with oseltamivir and suggested that higher doses of oseltamivir may be needed (*1*,*8*). Because the clade 2 viruses studied here have a 15- to 30-fold decrease in sensitivity compared to the clade 1 viruses, this suggests the standard dosing of oseltamivir may be even less effective in treating clade 2 influenza (H5N1)–infected patients.

Many laboratories are developing rapid PCR sequencing methods for detecting the known mutation (H274Y) that confers high-level resistance in influenza (H5N1) viruses. However, we have shown here the importance of phenotypic testing of isolates in an enzyme assay rather than just genotypic screening (*14*). Because the clade 2 virus is now spread through parts of Europe and Africa, continued global collaboration and phenotypic testing of drug sensitivity of circulating highly pathogenic avian isolates for NA inhibitor sensitivity are critical. This knowledge is essential for developing appropriate management strategies for pandemic planning. No altered sensitivity to zanamivir occurred, which further supports the hypothesis of minimalist drug design (*15*) and of maintaining the inhibitor as close as possible to the natural substrate to minimize the emergence of resistance. Our results suggest that zanamivir may also play an important role in pandemic stockpiles.

## Footnotes

1 Isolates tested were the following: A/Mississippi/3/2001 wt, A/Mississippi/3/2001 H274Y; A/quail/Kelantan/6309/2004, A/chicken/Kelantan/5858/2004, A/quail/Vietnam/007B/2004, A/duck/Vietnam/007A/2004, A/chicken/Vietnam/0024/2004, A/chicken/Vietnam/0023/2004 A/chicken/Vietnam/0018/2004, A/chicken/Vietnam/0015/2004, A/chicken/Vietnam/0010/2004, A/chicken/Vietnam/008/2004; A/chicken/Cambodia/Siem Reap/77B/2004, A/chicken/Cambodia/Siem Reap/76/2004, A/chicken/Cambodia/Takeo/45/2004, A/chicken/Cambodia/Kandal/23/2004, A/chicken/Cambodia/Pong Peay/1B/2004, A/chicken/Cambodia/Pong Peay/1A/2004, A/goose/Cambodia/Kandal/2005, A/chicken/Cambodia/Kandal/3/2005, A/chicken/Cambodia/Kandal/2/2005, A/chicken/Cambodia/Kandal/1/2005, A/chicken/Indonesia/Wates/130/2005, A/chicken/Indonesia/Wates/126/2005, A/chicken/Indonesia/Wates/83/2005, A/chicken/Indonesia/Wates/80/2005, A/chicken/Indonesia/Wates/77/2005, A/chicken/Indonesia/Wates/1/2005.

## References

[1] De Clercq E, Neyts J. Avian influenza A (H5N1) infection: targets and strategies for chemotherapeutic intervention. Trends Pharmacol Sci. 2007;28:280–5. 10.1016/j.tips.2007.04.005 17481739 PMC7112898

[2] McKimm-Breschkin JL, Blick TJ, Sahasrabudhe A, Tiong T, Marshall D, Hart GJ, Generation and characterization of variants of NWS/G70C influenza virus after in vitro passage in 4-amino-Neu5Ac2en and 4-guanidino-Neu5Ac2en. Antimicrob Agents Chemother. 1996;40:40–6. 8787876 10.1128/aac.40.1.40 PMC163053

[3] Wetherall NT, Trivedi T, Zeller J, Hodges-Savola C, McKimm-Breschkin JL, Zambon M, evaluation of neuraminidase enzyme assays using different substrates to measure susceptibility of influenza virus clinical isolates to neuraminidase inhibitors: report of the neuraminidase inhibitor susceptibility network. J Clin Microbiol. 2003;41:742–50. 10.1128/JCM.41.2.742-750.2003 12574276 PMC149673

[4] Rameix-Welti MA, Agou F, Buchy P, Mardy S, Aubin JT, Veron M, Natural variation can significantly alter sensitivity to oseltamivir of influenza A (H5N1) viruses. Antimicrob Agents Chemother. 2006;50:3809–15. 10.1128/AAC.00645-06 16940075 PMC1635199

[5] Govorkova EA, Ilyushina NA, Boltz DA, Douglas A, Yilmaz N, Webster RG. Efficacy of oseltamivir therapy in ferrets inoculated with different clades of H5N1 influenza virus. Antimicrob Agents Chemother. 2007;51:1414–24. 10.1128/AAC.01312-06 17296744 PMC1855473

[6] Hurt AC, Selleck P, Komadina N, Shaw R, Brown L, Barr IG. Susceptibility of highly pathogenic A(H5N1) avian influenza viruses to the neuraminidase inhibitors and adamantanes. Antiviral Res. 2007;73:228–31. 10.1016/j.antiviral.2006.10.004 17112602

[7] World Health Organization. Tamiflu resistance found in Egypt patients. Press release 2007 Jan 22. [cited 2007 Feb 2]. Available from http://www.emro.who.int/csr/media/pdf/ai_press_22_01_07.pdf

[8] Le QM, Kiso M, Someya K, Sakai YT, Nguyen TH, Nguyen KH, Avian flu: isolation of drug-resistant H5N1 virus. Nature. 2005;437:1108. 10.1038/4371108a 16228009

[9] McKimm-Breschkin JL. Management of influenza virus infections with neuraminidase inhibitors: detection, incidence, and implications of drug resistance. Treat Respir Med. 2005;4:107–16. 10.2165/00151829-200504020-00004 15813662 PMC7099216

[10] Russell RJ, Haire LF, Stevens DJ, Collins PJ, Lin YP, Blackburn GM, The structure of H5N1 avian influenza neuraminidase suggests new opportunities for drug design. Nature. 2006;443:45–9. 10.1038/nature05114 16915235

[11] Monto AS, McKimm-Breschkin JL, Macken C, Hampson AW, Hay A, Klimov A, Detection of influenza viruses resistant to neuraminidase inhibitors in global surveillance during the first 3 years of their use. Antimicrob Agents Chemother. 2006;50:2395–402. 10.1128/AAC.01339-05 16801417 PMC1489772

[12] Zurcher T, Yates PJ, Daly J, Sahasrabudhe A, Walters M, Dash L, Mutations conferring zanamivir resistance in human influenza virus N2 neuraminidases compromise virus fitness and are not stably maintained in vitro. J Antimicrob Chemother. 2006;58:723–32. 10.1093/jac/dkl321 16891631

[13] Kiso M, Mitamura K, Sakai-Tagawa Y, Shiraishi K, Kawakami C, Kimura K, Resistant influenza A viruses in children treated with oseltamivir: descriptive study. Lancet. 2004;364:759–65. 10.1016/S0140-6736(04)16934-1 15337401

[14] Smith GJ, Fan XH, Wang J, Li KS, Qin K, Zhang JX, Emergence and predominance of an H5N1 influenza variant in China. Proc Natl Acad Sci U S A. 2006;103:16936–41. 10.1073/pnas.0608157103 17075062 PMC1636557

[15] Varghese JN, Smith PW, Sollis SL, Blick TJ, Sahasrabudhe A, McKimm-Breschkin JL, Drug design against a shifting target: a structural basis for resistance to inhibitors in a variant of influenza virus neuraminidase. Structure. 1998;6:735–46. 10.1016/S0969-2126(98)00075-6 9655825

